# Proteinaceous Regulators and Inhibitors of Protein Tyrosine Phosphatases

**DOI:** 10.3390/molecules23020395

**Published:** 2018-02-12

**Authors:** Wiljan Hendriks, Annika Bourgonje, William Leenders, Rafael Pulido

**Affiliations:** 1 Department of Cell Biology, Radboud University Medical Center, Geert Grooteplein 26, 6525 GA Nijmegen, The Netherlands; annika.bourgonje@radboudumc.nl; 2 Department of Biochemistry, Radboud University Medical Center, Geert Grooteplein 26, 6525 GA Nijmegen, The Netherlands; william.leenders@radboudumc.nl; 3 Biomarkers in Cancer Unit, Biocruces Health Research Institute, 48903 Barakaldo, Spain; 4 IKERBASQUE, Basque Foundation for Science, 48011 Bilbao, Spain; rpulidomurillo@gmail.com

**Keywords:** biologics, PDZ domain, protein inhibitors, protein tyrosine kinases, protein–protein interaction, regulation of activity, signal transduction

## Abstract

Proper control of the phosphotyrosine content in signal transduction proteins is essential for normal cell behavior and is lost in many pathologies. Attempts to normalize aberrant tyrosine phosphorylation levels in disease states currently involve either the application of small compounds that inhibit tyrosine kinases (TKs) or the addition of growth factors or their mimetics to boost receptor-type TK activity. Therapies that target the TK enzymatic counterparts, the multi-enzyme family of protein tyrosine phosphatases (PTPs), are still lacking despite their undisputed involvement in human diseases. Efforts to pharmacologically modulate PTP activity have been frustrated by the conserved structure of the PTP catalytic core, providing a daunting problem with respect to target specificity. Over the years, however, many different protein interaction-based regulatory mechanisms that control PTP activity have been uncovered, providing alternative possibilities to control PTPs individually. Here, we review these regulatory principles, discuss existing biologics and proteinaceous compounds that affect PTP activity, and mention future opportunities to drug PTPs via these regulatory concepts.

## 1. Introduction

Cells integrate internal and external signals into appropriate adaptive responses. This signal processing should be timely and efficient, both from an economic and logistic point of view. Cells cannot rely on time-consuming de novo synthesis of RNA and protein components when fast responses are needed. Rather, they make use of a plethora of—often reversible—ways to modify biomolecules. One of the most abundant and best studied posttranslational protein modifications in signal transduction is reversible phosphorylation of serine, threonine and tyrosine residues. Protein phosphorylation is key in virtually all cellular processes [1], and especially phosphotyrosine-dependent events are associated with signaling networks that drive proliferation and differentiation of cells in multi-cellular organisms. Therefore, a considerable part of the hereditary and acquired diseases in humans are caused by defects in phosphotyrosine signaling relays [2,3]. 

The specific phosphorylation status of proteins within cells is tightly controlled by opposing kinase and phosphatase activities. Protein tyrosine kinases (TKs) add phosphate groups to selected tyrosine residues on proteins. Vice versa, members of the highly conserved protein tyrosine phosphatase (PTP) family dephosphorylate these phosphotyrosine sites. One should not conclude, however, that PTPs merely oppose TK functions; multiple examples exist that demonstrate that TKs and PTPs may also act in concert. Most phosphorylation events do not just target a single signaling pathway in the cell but have modulating effects that spread across multiple interconnected networks, thereby changing signaling specificity and outcome. Generally speaking, kinases are thought to control the amplitude of a certain signaling response and phosphatases are rather determining the response rate and duration [4,5]. 

**Figure 1:**
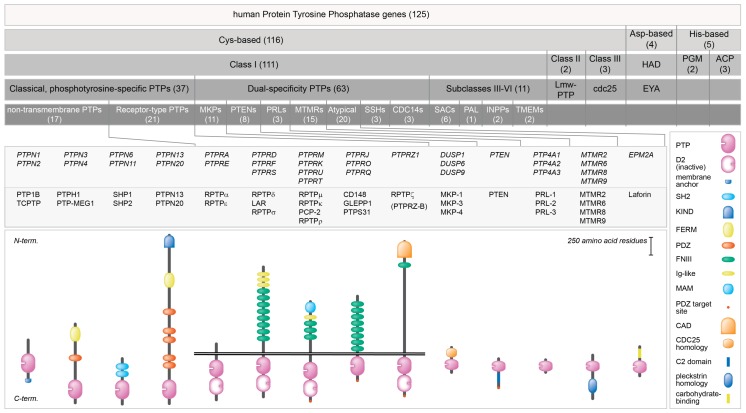
Classification and protein domain structure of PTPs discussed in this manuscript. Top five bars summarize the family’s (sub)class build-up, with number of genes indicated in between brackets. In the lower part, gene (italics) and protein names of representative PTPs, as well as the structures of fourteen PTP types are given. Horizontal twin black lines represent the plasma membrane. Protein domain representations are explained on the right. CAD, carbonic anhydrase-like; C2, protein kinase C conserved region; D2, inactive PTP domain; FERM, 4.1 protein-ezrin-radixin-moesin; FNIII, Fibronectin type three; Ig-like, Immunoglobulin-like; KIND, kinase non-catalytic C-lobe; MAM, meprin, A-5 protein, and RPTP-mu; PDZ, postsynaptic density-95/discs large/ZO1 homology; PTP, catalytic domain; SH2, Src Homology 2. Based on [2,6].

PTPs are evolutionary well preserved proteins that share a conserved region in their catalytic site (CX_5_R) containing an essential cysteine residue, which is responsible for removing the phosphate group from phosphorylated tyrosine residues. PTPs can be classified into several classes. Class I PTPs represent the largest group in which a distinction can be made between cytosolic non-receptor PTPs, transmembrane receptor PTPs (RPTPs) and dual-specificity PTPs (DUSPs). The dual-specificity subgroup can be further subdivided into MAPK phosphatases (MKPs), PTEN-related phosphatases (PTENs), phosphatases of regenerating liver (PRLs), myotubularin-related phosphatases (MTMRs) and atypical PTPs. The remaining cysteine-based classes are much smaller and consist of low-molecular weight (lmw) PTP (class II) and cell division cycle (cdc25) (class III). There is molecular evolutionary and enzymatic evidence to also include the haloacid dehalogenase (HAD), phosphoglycerate mutase (PGM) and acid phosphatase (ACP) families as PTPs. Instead of cysteine, these enzymes exploit aspartate (HAD) or histidine (PGM and ACP) in their PTP active site. In total, this brings the human PTP superfamily to the level of 125 protein-encoding genes [6] (Figure 1).

When analyzing the gene build-up within the PTP superfamily, it becomes apparent that nature invented these enzyme machines several times independently, but in all cases ended up with a so-called P-loop that harnesses the active site residue and a general acid/base-containing WPD loop involved in substrate binding. The evolutionary history of involved genes thus represents an impressive example of convergent evolution [7,8]. The PTP active site is formed by a 0.5–1.0 nm deep cleft that is optimized to accommodate the substrate’s phospho-residue and to facilitate its dephosphorylation. The latter occurs via a two-step reaction that involves: (i) the formation of a covalent phosphoryl enzyme intermediate; and (ii) the subsequent water-mediated hydrolysis to release the phosphate ion from the enzyme. This very conserved efficient overall catalytic mechanism has rendered the design of specific catalytic inhibitors for individual family members to be very difficult [9,10]. Not surprisingly, the protein phosphatases family was not represented in Hopkins and Groom’s overview of the druggable genome [11] but recent developments challenge their stigma of being undruggable [12]. Other contributions in this Special Issue highlight steps and breakthroughs in the research towards small-molecule inhibitors for PTPs. Here we will concentrate on the development of protein-based drugs that modulate PTP activity by interfering with protein–protein interactions (PPIs). Before doing so we will first briefly introduce the PPI-based regulatory principles that steer PTP action. 

## 2. Inhibiting and Augmenting PTP Activities

Two discoveries launched major pharmaceutical attempts to search for PTP inhibitors: the finding that PTP1B knockout mice are insulin hypersensitive and lost the ability to become obese [13,14] and the notion that *SHP2* classified as an oncogene [15]. Envisaged small-molecule inhibitors were meant to block enzyme activity in order to treat type 2 diabetes and obesity or cancer. As mentioned, the conserved structure of the PTP domain poses a considerable problem regarding drug specificity. To circumvent this hurdle, antisense oligonucleotide treatment to reduce protein—and thus activity—levels have been explored in mice for PTP1B [16] and lmwPTP [17]. The difficulty of in vivo delivery of antisense oligonucleotides, however, has led to continuing efforts to generate small-compound inhibitors, and clinical trials with drugs that display sufficient specificity towards VEPTP [12], PTP1B and SHP2 [18] are now underway. 

**Figure 2:**
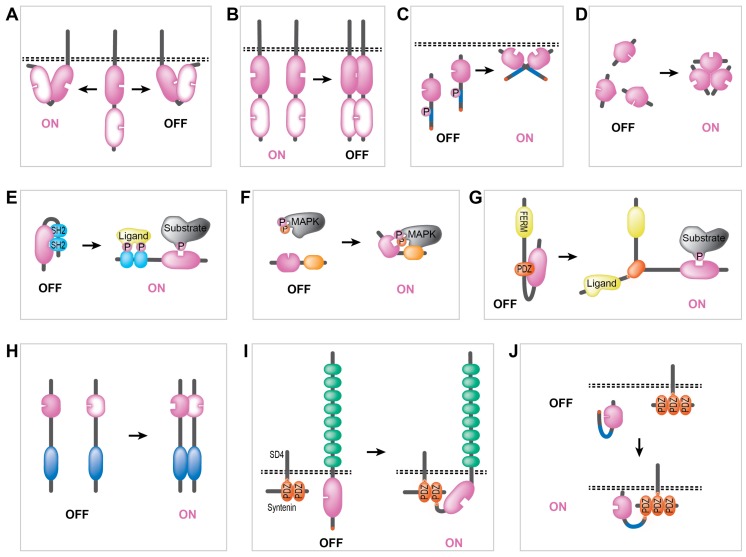
Protein interaction-dependent regulatory mechanisms impinging on PTPs. (**A**) Intramolecular interactions between membrane-proximal and -distal PTP domains may boost or attenuate enzymatic activity. (**B**) Dimerization of RPTPs usually inhibits enzyme activity. (**C**) Active PTEN homodimers form once the C-terminal part is dephosphorylated. (**D**) PRLs trimer formation enables enzyme activity. (**E**) SH2-containing PTPs are activated by phosphotyrosine-containing ligands. (**F**) Interactions between the CDC25-like domain in MAP kinase phosphatases and the substrate MAPK boost PTP activity. (**G**) PDZ target sequences, upon binding to PDZ domains in PTPs, trigger enzymatic activity of the latter. (**H**) Heterodimerization with inactive members of the MTMR subfamily boosts the enzymatic function of active subfamily members. (**I**) Submembranous PDZ domains trigger enzymatic activity upon binding to PDZ target sequences in RPTPs. (**J**) Submembranous complexes with PDZ-containing proteins are able to recruit the activity of PDZ target sequence-containing PTPs. For more details on these types of regulation and the associated references we refer to the text.

There are multiple PTPs for which the signaling role rather fits the tyrosine kinase opposing, tumor suppressive description. Therapeutic intervention then requires the development of drugs that boost the activity of specific PTPs. Replenishment of PTP activity is also needed following their transient oxidation and inactivation resulting from the local peak in intracellular H_2_O_2_ production that parallels physiological phosphotyrosine-based signal transduction [19]. This redox sensitivity of PTP enzymes is attributable to the build-up of the active site, in which the essential cysteine residue experiences a slightly acidic environment and is predominantly in the thiolate form. This serves well as a nucleophile in the two-step dephosphorylation reaction mechanism that characterizes PTPs but it also causes oxidation vulnerability. Many PTPs appear constitutively active when studied in isolation but evidence is accumulating that they are often regulated by intra- and intermolecular interactions. Increased knowledge of such regulatory mechanisms will ultimately allow the development of therapeutics, be it small-molecule or biomolecule-derived compounds, that enable the fine-tuning of specific PTP activities at will. Some PTPs demonstrate impressive protein–protein anchoring and scaffolding potential and therefore PTP activity may extend beyond enzymatic handling of phosphotyrosine-containing proteins. An example is provided by the “moonlighting” activities of the well-known tumor suppressor protein phosphatase and tensin homolog (PTEN) that include protein phosphatase as well as lipid phosphatase activity, and, in addition, non-catalytic nuclear roles in supporting chromosomal stability and maintenance of genome integrity [20,21]. While reviewing the protein interaction-based PTP regulatory mechanisms (see also Figure 2), we will therefore mention those that impact on enzyme activity and the ones that impinge on the protein–protein interactive capacities.

### 2.1. Intra-Molecular (Allosteric) Regulation of PTP Activity

The proto-oncogene *PTPN11* encodes SHP2, a ubiquitously expressed cytosolic PTP that has its enzymatic phosphotyrosine phosphatase domain N-terminally preceded by two SH2 domains. SHP2 is in an inactive state when its N-terminal SH2 domain folds onto the PTP domain, thereby blocking the PTP active site and preventing substrate binding (Figure 2E). Following growth factor receptor activation specific phosphotyrosine motifs in the receptor TKs and in their substrates, including docking adapter proteins, will lure the SHP2 N-terminal SH2 domain away from the PTP domain, unleashing the domain’s activity [22]. *PTPN11* mutations are found in patients with Noonan Syndrome (NS), Leopard Syndrome (LS), juvenile myelomonocytic leukemia (JMML), acute myelogenous leukemia (AML) and various solid tumors [2]. At the protein level, these mutations disrupt the allosteric interaction and cause SHP2 to be constitutively active [23]. Thus, inhibiting SHP2 activity is a promising strategy to treat various cancers. Apart from drugs directly aiming at the active site, indeed small-molecule SHP2 inhibitors that in fact “glue” the enzyme in its inactive conformation have been developed [24,25,26]. 

A special group of dual specificity phosphatases (DUSPs) act as mitogen-activated protein kinase phosphatases (MKPs) [27], and for some of the members, such as MKP-3 (encoded by the *DUSP6* gene), firm proof of a substrate-induced allosteric regulatory mechanism has been provided [28,29,30]. MKPs all have a C-terminal PTP domain, an N-terminus that dictates subcellular localization and in between there is a kinase interaction motif (KIM) that determines MAPK recognition (Figure 2F). MKP3 has a relatively high substrate specificity; it dephosphorylates the activation loop phosphothreonine and phosphotyrosine residues in ERK1/2 [31]. MKP3 displays low basal enzymatic activity but KIM-mediated binding to its substrate induces a repositioning of key active-site residues that likely facilitate WPD loop closure, boosting its activity and explaining its substrate specificity [28,29,30]. Based on this mechanism, allosteric inhibitors of MKP3 have been developed [32,33].

Most receptor-type PTPs (RPTPs) display tandem phosphatase domains in their intracellular portion; the enzymatic activity is primarily attributable to the membrane proximal domain (D1), while the membrane distal domain (D2) usually is inactive [34]. It is still a conundrum how the D2 domain contributes to RPTP function. Suggestions converge on regulating D1’s substrate specificity and/or enzyme activity (Figure 2A). An intriguing example is found in the comparative study on two *Drosophila* RPTPs, DLAR and PTP99A [35]. Using bacterially produced recombinant RPTP segments in activity and binding assays and subsequent in silico structural studies it was found that the DLAR D2 domain decreased the D1 activity via an inter-domain interaction whereas the PTP99A D2 domain caused an increase in the D1 activity. If and how the linker regions, which connect D1 and D2, are involved in mediating these allosteric effects in DLAR and PTP99A [36] requires further investigation.

### 2.2. Inter-Molecular Regulation of PTP Activity

Receptor-type TKs become active once ligand binding triggers their dimerization. In contrast, RPTPs present as inactive homodimers [37,38] or heterodimers [39] on the cell surface. It remains to be established whether and how ligand binding is involved (Figure 2B). Structural studies initially suggested that a sequence motif termed “wedge domain”, located between the transmembrane region and the D1 domain, is responsible for dimer-induced RPTP inactivation by occluding the catalytic center of D1 in the partner RPTP [40,41,42]. In addition, the activity of RPTPs that do not contain such a wedge domain was found to be reduced upon dimerization (e.g., [43]). As yet, only a limited number of bona fide RPTP ligands have been identified [44], and for PTPRZ-B the inhibitory effect of its ligand pleiotrophin on activity has been shown [45,46]. In line, dissociation of the dimeric RPTP SAP-1 by a reducing environment results in increased enzymatic activity of SAP-1 monomers [47].

Not only transmembrane PTPs form multimers, also some non-receptor type family members present as oligomers. This was first demonstrated for a naturally occurring cytoplasmic variant encoded by the RPTP gene *PTPRE* [48]. Dimerization tendency of this cyt-PTP epsilon isoform appeared the net result of the impact of its inactive D2 domain, that supported a strong intermolecular interaction inhibiting cyt-PTP epsilon activity, versus effects by D2 flanking sequences that prevented constitutive dimerization and inactivation [48]. Likewise, the *DUSP3* gene product, VHR, forms transient homodimers inside cells, resulting in a temporal inactivation of the dual-specificity phosphatase [49]. In addition, laforin, the Lafora disease-associated PTP that dephosphorylates branched glucose polymers, is able to form dimers or even multimers, but here oligomerisation of the enzyme results in cooperative substrate binding and increased activity [50]. The PTEN tumor suppressor’s phosphatase activity on PI(3,4,5)P_3_ also depends on dimerization (Figure 2C), which requires its C-terminal tail to be unphosphorylated [51,52]. Likewise, for the myotubularin-related (MTMR) 14-member subfamily of PTPs, enzyme activity towards phosphoinositides is regulated via homo- and hetero-dimer formation (Figure 2H). MTMR2, for example, requires the formation of membrane-associated dimers to exert its role in PI(3,5)P_2_ and PI(3)P turnover [53]. Phosphatase-inactive MTMR9 is able to increase enzymatic activity, and even substrate specificity, of MTMR6 and MTMR8 via heterodimerization [54,55]. Finally, the phosphatase of regenerating liver (PRL) phosphatases, that are able to stimulate ERK1/2 and Akt signaling and are overexpressed in many cancer types [56], also depend on oligomerization for their activity. Here, not dimers but trimers represent the active conformation of the enzymes (Figure 2D). In line with this, a small compound, termed Cmpd-43, that prevented PRL1 trimer formation indeed successfully abrogated the enzyme’s activity [57].

### 2.3. Regulating Anchoring and Scaffolding Activity of PTPs

Although the paradigm activity of PTPs is the dephosphorylation of proteins—or of other biomolecules including phosphoinositides [58], glycogens and even RNA—several PTP family members are catalytically inactive [6]. As observed for the MTMR subfamily, such inactive representatives are well capable of regulating the activities of other PTPs. In addition, RPTP ligands serve as examples of proteins that modulate PTP activity by means of protein–protein interactions. However, there are additional PTP binders that need to be introduced here. Proteins harboring so-called PDZ (PSD-95, Dlg, ZO-1) domains have demonstrated unprecedented powers in orchestrating submembranous signaling complexes [59] and in doing so they regulate the stability and activity of transmembrane receptors (e.g., [60]) as well as cytoplasmic proteins. For example, amyloid-beta aggregates, which lead to synaptic toxicity and cognitive dysfunction in Alzheimer’s disease, initiate the recruitment of PTEN to the postsynaptic area in brain cells. This PTEN relocation was dependent of its C-terminal PDZ target site (Figure 2J), as demonstrated by the use of a cell-permeable interfering peptide [61]. Remarkably, non-peptidic small molecules mimicking PDZ-binding motifs are proficient to inhibit the interaction of PTEN with PDZ domains [62,63], indicating their potential as scaffolds for therapeutic drug development.

In a study showing how syndecan-4 (SD4) is able to down-regulate T-cell receptor (TCR)-mediated signals coming from the immune synapse, again a crucial contribution of PDZ domain-mediated interactions was disclosed [64]. The immune receptor SD4 is expressed on activated T-cells and its C-terminus is permanently bound by one of syntenin’s tandem PDZ domains. Upon ligand binding to SD4, syntenin additionally binds to and activates CD148 [64], a receptor-type PTP encoded by gene *PTPRJ* (Figure 2I), likely through a second PDZ domain mediated interaction [65]. Another fine example comes from PTPN4, a cytosolic PTP that consists of an N-terminal FERM domain, a middle segment harboring a single PDZ domain, and C-terminally the catalytic PTP domain (Figure 2G). PTPN4 normally protects cells from apoptosis but addition of ligands for the PTPN4 PDZ domain resulted in a marked induction of cell death [66]. Subsequent studies revealed that the PTPN4 PDZ domain in fact auto-inhibits the enzyme’s catalytic activity, and this is abrogated upon binding of a PDZ domain ligand [67,68]. The segment linking the PDZ and PTP domain in PTPN4 is also contributing to this regulatory principle [69,70]. Thus, possibilities to interfere with PDZ-mediated PPIs represent novel routes to correct disease-related signaling processes.

## 3. Expanding the Druggable Genome Using Proteinaceous Drugs

From the above, the picture emerges that novel possibilities to modulate PTP activities can be envisioned when focus is redirected from the catalytic core towards enzyme parts that are involved in regulatory PPIs. For long, the field has been reluctant towards the development of drugs that would interfere with PPIs but slowly the tide is turning [71,72]. For example, venetoclax, a drug that prevents the anti-apoptotic BCL-2 protein to interact with pro-apoptotic BAX and BAK, has successfully entered the clinic for treatment of lymphoid malignancies [73]. We already mentioned small compound PPI inhibitors that successfully inactivate SHP2 and PRL1 by targeting intra- and intermolecular interactions, respectively [24,25,57], and thus opt for potential cancer therapeutics [74]. Not surprisingly, also PDZ-mediated interactions gained quite some attention as potential drug targets for PPI inhibitor design. Based on the C-terminal peptide sequence from one of the ligands for the human AF6 (ALL1-fused gene from chromosome 6) PDZ domain, for example, high-affinity small compounds have been produced that inhibit AF6-Bcr interactions and interfere with growth factor signaling in leukemia cells [75]. Furthermore, compounds ZL006 and IC87201, identified as efficient inhibitors of the PDZ-based nNOS/PSD-95 interaction, are being considered as promising drugs to treat ischemic stroke and pain [76]. Confining ourselves to PTPs as drug targets, the findings from studies on idiopathic pulmonary fibrosis come to mind [77] which led to the patenting of a small molecule that blocks the PDZ-mediated association of PTPN13 with the C-terminus of FAS (US Patent Application 20120148528; Published international patent WO/2012/064763).

**Figure 3:**
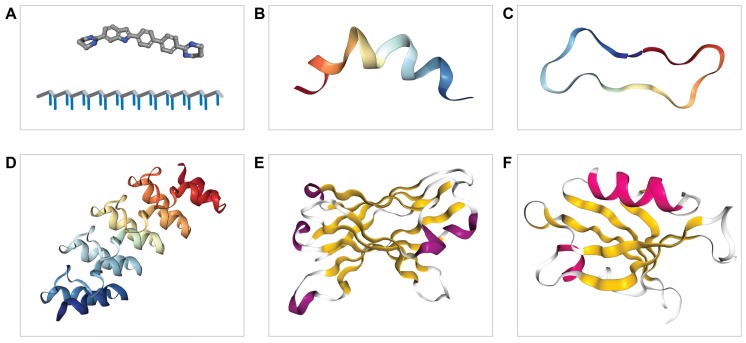
Approaches to interfere with protein–protein interactions: (**A**) small compounds (upper) or RNAi-based drugs (lower); (**B**) linear interfering peptides; (**C**) stabilized, (bi)cyclic peptides; (**D**) scaffolds; (**E**) protein fragments; and (**F**) protein domains. Images are based on PDB-derived structures of DNA-binding compound DB1804 (A: 3U05), cell penetrating peptide Penetretin (B: 2ND6), one half of a sunflower trypsin inhibitor-1-derived, backbone-cyclized disulfide-bridged 16-mer peptide (C: 2BEY), a tubulin-binding Darpin (D: 5NQU; binding interface is formed by the lower-right loops), the single chain antibody fragment scA21 against ErbB2 (E: 2GJJ; antigen binding site is on the left), and the second PDZ domain in PTPN13 (F: 1GM1; the ligand, a C-terminal PDZ target site, will bind in the groove formed by the top alpha helical structure and the beta strand that runs parallel).

Given that the protein surfaces involved in PPIs are usually much larger than the catalytic pockets targeted by small molecule inhibitors, this novel playground holds great promise for larger, protein-based compounds (Figure 3). Although for intracellular PPI targets the design of small molecule inhibitors is more obvious in view of delivery, PPI inhibitor discovery for extracellular interactions is also open for proteinaceous approaches [78]. These include antibody-based and protein- and peptide-display library strategies [79] and resulting tools are then indicated with names like single-chain variable fragments, darpins, monobodies or foldamers. In addition, peptide- and peptidomimetics-based PPIs are explored. As opposed to small chemical therapeutics, the development of such proteinaceous drugs faces distinct challenges regarding immunogenicity, proteolytic sensitivity and tissue and cell penetrability, but promising developments in these areas are penetrating the literature. We will briefly introduce the basics underlying these protein-based tools before turning to their applications within the PTP field.

The most obvious approach to design a PPI inhibitor, i.e., pick a peptide sequence derived from the interface of the target PPI, actually appears quite effective despite the fact that relevant 3D protein structures—let alone spatial information on the protein–protein interactions themselves—are usually lacking. Homology modeling and dynamic calculations may help realize a computational design. Alternatively, a functional approach such as display library screening could serve in yielding a peptide starting point [72,78]. The next hurdle is then impinged by the alleged folding structure; does the peptide reflect an alpha-helix, a beta hairpin or rather a coiled segment? Quite often protein interaction interfaces involve alpha-helical structures [80] but since there is a large entropic penalty for binding, a stabilization of peptide structures seems mandatory. Unmodified peptides in isolation appear poor inhibitors, and the success of peptide display screening strategies mirrors just that; peptides are then presented within a protein domain context. Researchers have therefore explored possibilities to stabilize peptide segments through modifications, usually cyclization or stapling [81]. In addition, the synthesis of peptide-like molecules that mimic protein structural segments, carrying names like peptoids or foldamers, and display improved biostability and cell permeability is being pursued [82,83]. Especially the possibility to conjugate peptides or peptidomimetics with other molecules opens new avenues for peptide therapeutics development. Dozens of peptide conjugates are currently being evaluated in clinical trials. The combination of a peptide component and a chemotherapeutic agent, for example, may ensure targeted delivery at the relevant site, merging improved safety and higher dosing of the anticancer agent [84]. As structural information on PPIs and the chemist’s toolbox to produce peptidomimetics are expanding [85], it is a matter of time before such compounds make up a considerable part of the peptide therapeutics that enter the clinic [86,87].

Zooming out from peptide(-like) molecules that modulate PPIs (Figure 3A–C), the next option would be to turn to larger peptide scaffolds (of some 20–50 amino acid residues), protein domains or even whole proteins. After all one could view such larger structures as composed of two distinct regions; a core scaffold fused to a bioactive peptide sequence or peptide-binding structure (Figure 3D–F). Many different examples have been described in the literature, ranging from decoy receptors that abrogate ligand–receptor binding [88] to antibody-derived fragments such as immunoglobin Fc domains [89] or single-chain variable fragments (scFvs) [90]. Decoy receptors result from proteolytic cleavage of genuine receptors and/or alternative splicing of receptor-encoding transcripts [88] and are of interest because of their non-immunogenicity in humans. Immunoglobulin-derived molecules make up a major part of the current-day biologics used in the clinic [91], but also many non-immunoglobulin-based protein domains and scaffolds, such as monobodies [92] and darpins [93], have been explored in the search for specific, stable and bioactive binding modules that target proteins [91,92]. Some of these tools are indeed exploited to develop PTP inhibitors, as described below. 

## 4. Towards Proteinaceous Drugs that Regulate Protein Tyrosine Phosphatases

**Figure 4:**
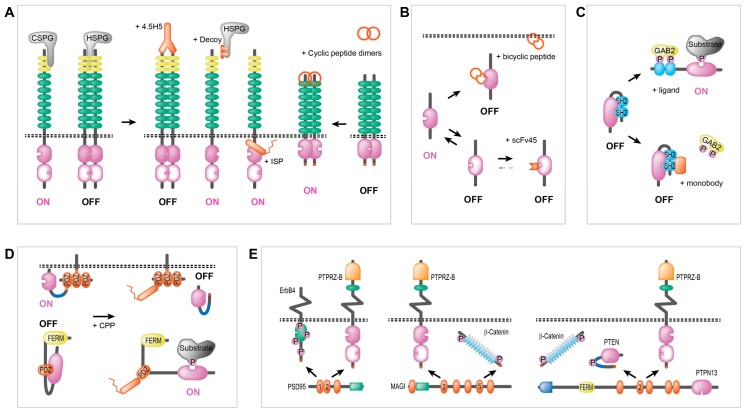
Current examples of proteinaceous PTP modulators. (**A**) Regulation of RPTPσ activity by CSPG or HSPG ligands, dimerizing antibodies (4.5H5), dimerization-blocking decoy receptors or a cell-penetrating “wedge” peptide (ISP). In addition, cyclic peptide dimers that activate CD148 are shown. (**B**) Inhibition of PTP1B activity by a bicyclic peptide containing a cell-penetrating and an enzyme-inhibiting peptide loop, or by an intracellular single-chain variable fragment (scFv45) that stabilizes the partly oxidized, inactive form of the enzyme. (**C**) Abrogation of the ligand-induced activation of SHP2 using a monobody that blocks SH2 domain interactions with the ligand. (**D**) Interfering with PDZ-mediated regulatory principles. Cell-penetrating peptides (CPP) prevent PTEN recruitment to the postsynaptic density (upper half) or activate PTPN4 in glioblastoma cells (lower part). (**E**) Schematic depiction of targetable submembranous PDZ-based interactions in glioblastoma cells. PTEN, PTPRZ-B, ErbB4 and β-catenin all can interact with PSD95, MAGI family members and PTPN13 PDZ domains. Associations may be mutually exclusive, as for PTEN and PTPRZ-B binding to PTPN13 PDZ2, or additive. The ErbB4 TK domain is in green, blue diamonds represent β-catenin armadillo repeats, and the green rectangle indicates the membrane-associated guanylate kinase domain in PSD95 and MAGI proteins. Other protein domains are explained in Figure 1. See the text for more details and references.

Ever since the notion that receptor-type PTP dimerization alters their enzymatic activity [40] the impact of compounds that bind to the extracellular, ligand-binding segment became of interest as tools to boost or inhibit RPTPs. Bona fide RPTP ligands have hardly been identified [44] but the paradigm example of pleiotrophin, that upon binding abrogates PTPRZ-B activity through dimerization [45,46], underscores the potential. In line, antibodies that bind to RPTPs on the cell surface indeed are able to regulate their function (Figure 4A). A recent example comes from work from the Tremblay group that investigated the possibility to modulate RPTPσ activity using previously identified ligands and novel monoclonal antibodies [94]. On cells, RPTPσ present as dimers in a manner that is independent of the intracellular wedge domain and rather results from extracellular homotypic interactions [95,96]. Intriguingly, two ligands of RPTPσ—chondroitin sulfate and heparan sulfate proteoglycans (CSPGs and HSPGs)—compete for binding to the same Ig-like domain in the RPTPσ extracellular part but induce opposing effects; CSPGs prevent dimerization and HSPGs facilitate RPTPσ dimer formation [97,98]. A similar type of opposing regulation by proteoglycans had been noted previously for *Drosophila* LAR [99]. The RPTPσ-specific monoclonal antibody 4.5H5 was shown to firmly increase RPTPσ dimerization, translating in enhanced neuritogenesis in neurite outgrowth assays [94]. This provides proof of principle that such dimer-inducing, inactivating anti-RPTP antibodies (see Figure 4A) could be turned into biologics, for example for the treatment of patients with spinal cord injuries.

Antibodies may also be used to prevent PTP oligomerization. The notion that trimer formation is required for PRL enzymatic activity [57] suggests the possibility to design antibodies that would prevent PRL trimer formation. Although anti-PRL antibodies have been generated and proved valuable tools to treat PRL over-expressing cancers in preclinical settings [100,101,102], their mechanism of action is not preventing PRL trimers to form but rather mounting an efficient and specific antibody-mediated immune response against this intracellular oncotarget. As an alternative approach to prevent PTP oligomerization the exploitation of a decoy receptor has been reported [103]. In joint-lining synoviocytes, that in rheumatoid arthritis are responsible for joint inflammation and destruction of cartilage and bone, the HSPG syndecan-4 (SD4) constitutively interacts with—and clusters and inactivates—RPTPσ. Disruption of this interaction could be achieved by the extracellular provision of competing amounts of the ligand-binding RPTPσ Ig-like domain part, unleashing its intracellular enzyme activity. With this set-up, synoviocyte invasiveness and cartilage attachment was impaired, and established arthritis could be reversed in rheumatoid arthritis models [103].

Takahashi and coworkers generated a monoclonal antibody against the CD148 ectodomain on endothelial cells and demonstrated that it effectively blocked angiogenesis in mice. Contrary to what one might expect, this was not due to dimerization-induced inactivation of the RPTP, but rather an increased CD148 activity was noted [104]. Building on screening results from a combinatorial phage display library, Trapasso’s group optimized cyclic nonapeptides that bind to the CD148 ectodomain. These ligand mimetics had a high tendency to dimerize and were found to also activate the phosphatase [105]. Thus, RPTP oligomerization may impact differently on the catalytic activity depending on the individual components in the complex and the rotational coupling imposed upon the cytoplasmic moieties. This had been noted also in experiments addressing possible redox regulation of RPTPα dimers [106]. This redox sensitivity of PTP enzymes, observed as a local and transient PTP inactivation due to reactive oxygen species production that parallels growth factor signaling [19], actually allowed the development of a unique type of PTP biotherapeutic. The Tonks lab applied phage display technology to select a single-chain variable fragment, scFv45, that would specifically bind to the reversibly oxidized, sulfenyl-amide intermediate form of PTP1B that is catalytically inactive (PTP1B-OX) [107]. They hypothesized that such a conformation-specific antibody may stabilize the inactive state and thus inhibit PTP1B activity (Figure 4B). Using the conformation sensor scFv45 as “intrabody” indeed an attenuation of PTP1B activity in response to insulin-induced reactive oxygen species production and concomitant increased insulin receptor-dependent signaling was obtained [107], providing an alternative route to design PTP-specific inhibitors.

In addition, non-antibody scaffolds have been used to merge combinatorial library designs and selection strategies in order to isolate synthetic proteins that bind their targets with affinities and specificities that compare well to those of antibodies but come with advantages such as small size and lack of disulfide bonds [92]. A nice illustration comes from work on SHP2, the proto-oncogenic SH2 domain-containing cytosolic PTP that has been in the limelight as a drug target for quite a while [23] but again enzyme inhibitor development was frustrated by the conserved nature of the PTP domain [74]. Some years back, Sha et al. realized that also the SH2 domains in SHP2 pose a specificity problem due to their highly homologous nature [108]. Building on their success to generate highly specific monobodies, i.e., synthetic β-sandwich proteins based on the human fibronectin type III domain framework, that bind the BCR-ABL SH2 domain they screened for monobodies that would target the tandem SHP2 SH2 domains (Figure 4C). Indeed they obtained a set of tools that, when expressed in leukemic cells, differentially inhibit distinct roles of SHP2 in downstream signaling events, such as paxillin and STAT5 phosphorylation, depending on whether they competed with phosphotyrosine binding to the first or the second SH2 domain in SHP2 [108].

As mentioned, small-molecule inhibitor development for PTP1B had been frustrated due to the extreme resemblance of the active site in the paralogous T-cell PTP (TCPTP) [109]. Since the latter is linked to the development of multiple inflammatory diseases, therapeutic agents that selectively inhibit TCPTP would also be highly welcomed. Pei and coworkers took up the challenge to derive cell-permeable bicyclic peptidyl inhibitors for these closely related PTPs [110,111]. Their approach features a short L-2-naphthylalanine-containing cell-penetrating peptide in one ring and a target-binding peptide in the second (Figure 4B). They constructed a library of beads, with a complexity of 0.5 million, that each carried a random pentapeptide sequence with, on position 2, a phosphotyrosine mimic, and, on the other four positions, any amino acid out of a mixture of 10 proteinogenic and 5 unnatural l-amino acids, and 9 d-amino acids. Using PTP1B as bait for some 300,000 beads, 65 binders were identified and individually sequenced. Subsequent optimization resulted in a cell-permeable bicyclic peptidyl inhibitor for PTP1B (KD = 37 nM) that displayed a 17-fold selectivity over TCPTP [111]. Next, along similar lines, they mined for TCPTP-specific inhibitors. This time around one million compounds were affinity purified in two sequential rounds, yielding 137 positive beads. Following several optimization steps, the final bicyclic peptide inhibitor for TCPTP (IC_50_ = 110 nM) displayed effectiveness in EGF-stimulated C6 glioma cells but with only four-fold selectivity over PTP1B [110]. Overall, encouraging results that place the option of bicyclic peptides that combine cell-penetrating and target inhibiting characteristics as a potential strategy to produce PTP-regulatory compounds. However, current small-molecule inhibitors for these closely related PTPs appear much more potent and selective, and have even entered clinical trials [18].

Expanding on examples of peptide-based drugs, we return to the impact of the wedge domain on dimerization-regulated activity in a subset of receptor-type PTPs. Studies with RPTPα and CD45 had shown that such helix-loop-helix wedge domains block enzyme activity in RPTP homodimers [40,42]. The cell adhesion molecule-like receptor-type PTPs like LAR, RPTPµ and RPTPσ also contain a wedge domain in between their transmembrane and PTP domains [34] and “wedge peptides” have been designed in an attempt to interfere with RPTP functioning (Figure 4A). Cell-penetrating wedge peptides based on LAR and RPTPµ sequences have been tested in the rat PC12 cell model for NGF-induced neurite outgrowth or in retinal ganglion neurons seeded on a RPTPµ-exposing substrate, respectively [112]. Both “wedge Tat peptides” effectively and specifically blocked the survival- and neurite-promoting effects linked to their corresponding RPTPs [112]. Additional support for cell-penetrating wedge domain peptides as PTP inhibiting biologics comes from work on a cell-permeable wedge peptide drug for RPTPσ, termed “intracellular sigma peptide” (ISP). Silver and coworkers demonstrated its effectiveness in abrogating CSPG-RPTPσ-mediated inhibition of axonal regrowth and sprouting, hence facilitating functional recovery, in a spinal cord injury model [113]. Since CSPGs present in the cardiac scar after a myocardial infarction prevent sympathetic re-innervation by activating RPTPσ, they also studied the effects of daily injections of ISP in mice that experienced an experimentally induced infarction several days earlier. Application of the wedge peptide indeed restored sympathetic innervation, and the resulting infarcted hearts were electrically indistinguishable from un-infarcted hearts and resistant to induced arrhythmias [114]. In addition, in an animal model for severe nerve root injury, systemic delivery of ISP clearly enhanced nerve regeneration, firmly corroborating the therapeutic potential of this RPTPσ-specific cell-penetrating wedge peptide [115].

There is one particular regulatory part in PTPs that has not been discussed as a target of proteinaceous drugs, and that is the carboxyl-terminal region (Figure 4D). In a recent review [116] Elson and colleagues have compiled evidence that cytosolic tails of receptor-type PTPs are exploited in various ways for regulating phosphatase activity. Likewise, there is ample evidence that C-termini of cytosolic PTPs are impacting on enzyme behavior as well. This may reflect the PTP’s subcellular localization, as for e.g., PTP1B [117], but mostly these boil down to the protein–protein interactions mediated by so-called PDZ domains [118]. C-terminal PDZ domain binding sites have been noted in a considerable percentage of the RPTPs [116], but also in non-receptor PTPs like e.g., PTEN [119] such PPI-mediating C-termini are present. Adding to the complexity, several PTP family members (PTPN3, PTPN4, PTPN13 and PTPN20) actually contain PDZ domains themselves, shaping an extensive PPI network in control of reversible phosphotyrosine signaling in health and disease (Figure 4E). The PDZ domain-regulated impact of PTPN4 on glioblastoma cell survival [66] points to the modulation of PDZ-dependent PPIs as an attractive cancer treatment option. 

## 5. Untying Gordian Knots in Glioblastomas

Glioblastomas are brain tumors characterized by diffuse infiltrative growth in the surrounding parenchyma. Conventional therapeutic strategies combine surgical resection, radiotherapy and temozolomide chemotherapy [120] but as yet it remains impossible to cure patients, urging the need for increased knowledge on disease etiology to formulate novel therapeutic options. Some years back, we reviewed a list of PTP genes that bear links to gliomagenesis [121]. More recently, we classified a subset of PTPs as possible entry points for glioma treatment based on a comparison of their relative expression levels in normal and tumor brain material combined with expression and survival data from relevant databases [122]. Three of these genes (*PTPRZ1*, *PTEN*, *PTPRT*) encode PTPs with C-terminal PDZ binding motifs [116,123] and mapping of the interacting PDZ domain-containing proteins may be of relevance for glioblastoma etiology. 

*PTPRZ1* encodes three protein isoforms that share an N-terminal, extracellular carbonic anhydrase-like (CAH) and a fibronectin type III (FNIII) domain [124]. *PTPRZ1* expression, notably of the receptor-type PTPRZ-B isoform, is up-regulated in gliomas and several studies have underscored that down-regulation of PTPRZ-B reduces glioblastoma growth in in vivo models [125,126,127]. A peptide derived from PTPRZ-B’s extracellular moiety now forms part of a peptide-based glioma vaccine called IMA950 [128] that is currently in phase I/II clinical trials. Historically, a first in vivo attempt to therapeutically exploit PTPRZ overexpression in glioma dates back to 2006 when a saporin-coupled antibody against the PTPRZ-B extracellular domain was found to significantly delay human U87 glioma tumor growth in a subcutaneous mouse xenograft model [126]. The most recent endeavor reflects the development and testing of SCB4830, a small inhibitory compound that specifically targets the PTPRZ-B catalytic site [127]. Here, C6 glioblastoma cells had been transplanted into rat brains and the inhibitor was applied as a liposome complex by means of daily intracerebroventricular injections to find a reduction in tumor growth. 

While studying the role of PTPRZ-B in glioblastoma proliferation and migration we noted that its extracellular part is mediating migratory signals whereas the proliferative effect is exerted by the intracellular part [125]. More specially, the PTPRZ-B C-terminal PDZ binding motif appeared crucial for the latter process, and thus two PDZ domain-containing partners of PTPRZ-B need introduction: PSD95 and MAGI-2 (Figure 4E). PSD95 contains three PDZ domains of which the second binds to the PTPRZ-B C-terminus [129]. Using either its first or second PDZ domain PSD95 can also bind to ErbB4, a member of the broadly expressed family of epidermal growth factor receptor-type tyrosine kinases that impact on cell proliferation, survival and migration [130]. Thus, PSD95 can bind both proteins simultaneously, facilitating the dephosphorylation of ErbB4 by PTPRZ-B, but it can also facilitate ErbB4 homo-dimerization and subsequent auto-activation [131]. PDZ proteins thus may regulate phosphotyrosine-mediated signals through the organization of RTK/RPTP clusters. It also implies that changes in the expression of components participating in such regulatory clusters will have consequences. High PTPRZ-B levels, as in glioblastoma cells, thus may lead to decreased ErbB4 activity in two ways; by sequestering PSD95 and preventing PDZ-mediated ErbB4 homodimer formation as well as by directly dephosphorylating ErbB4. Unlike for its family member ErbB1 (EGFR), reduced ErbB4 activity rather facilitates proliferation, survival and differentiation of brain tissue (reviewed in [132]). It is tempting to speculate that the high PTPRZ-B levels in glioma, as part of their tumor-promoting effect, hamper ErbB4-mediated inhibition of proliferation. Proteinacious compounds that specifically block PDZ interactions have been derived, and these might prove beneficial in treating glioma. For instance, the peptide that blocks PDZ1 and PDZ2 in PSD95 [133] may disrupt the interaction with PTPRZ-B and affect glioblastoma cell proliferation.

PTPRZ-B and ErbB4 can also be bound by PDZ domains in the submembranous anchoring proteins MAGI-1, MAGI-2 and MAGI-3 [134]. MAGI-based ErbB4/PTPRZ-B complexes may serve similar purposes as described above for PSD95 (Figure 4E). Alternatively, PSD95 and MAGI could compete for binding to ErbB4 and PTPRZ-B and redirect them to different subcellular signaling niches. In addition, MAGI-1 serves as a PTPRZ-B substrate [135]. MAGI-3 is not a substrate itself but as a scaffold it provides PTPRZ-B access to phosphoproteins like p130 in glioma cells [136]. Likewise, MAGI-1 may facilitate β-catenin dephosphorylation by PTPRZ-B [45] through the recruitment of substrate and enzyme using its fifth and second PDZ domain, respectively [46,137] (Figure 4E). Of note, β-catenin levels are often upregulated in cancers and tyrosine-phosphorylated β-catenin increases Wnt signaling and tumorigenesis [138]. Since increased PTPRZ-B levels in the presence of abundant MAGI-1 should result in β-catenin dephosphorylation, hence tumor suppression, we reason that the MAGI–β-catenin link is unable to downplay PTPRZ-B’s tumorigenic role in glioma.

There are many more proteins that bind to PTPRZ-B [139] and in databases like BioGrid, String and InAct currently 138 are mentioned. This would provide a tremendous puzzle on how to keep PPI-disrupting compounds both targeted and effective. We found experimental evidence (our unpublished data) for yet another PTPRZ-B interacting protein, PTPN13 (Figure 4E). The handful of PDZ domains in this large submembranous protein enable PTPN13 to bind a wide range of proteins [140]. For example, PTPN13 PDZ domains bind the carboxyl-terminal “SLV” sequence in the programmed cell death receptor FAS [141]. In addition, PTPRZ-B ends in the “SLV” PDZ target sequence [116]. PTPN13 thus contributes to endocytosis and degradation of FAS, increasing cell resistance to apoptosis [141]. Conversely, cells with high autophagic activity degrade PTPN13 and become sensitive to FAS induced apoptosis [142]. In line, application of an “SLV” tripeptide or the functionally equivalent small molecule Quinobene in human CD34+ chronic myeloid leukemia cells increased their FAS sensitivity [143]. PTPN13 is an enigmatic player in the cancer field, displaying both oncogenic as well as tumor suppressive functions depending on tumor context [140]. For example, disruptive PTPN13 mutations have been found in various tumor samples [144,145] including HPV-negative head and neck squamous cell carcinomas [146]. In contrast, oncogenic functions for PTPN13 are suggested by an EWS-FLI1 chromosomal translocation in Ewing’s sarcoma that leads to a strong up-regulation of PTPN13 protein levels, thereby boosting cell growth and motility [147]. As mentioned above, PTPN13 facilitates resistance to apoptosis via the control of FAS cell surface levels (reviewed in [140]). Whether and how the PTPN13–PTPRZ-B interaction contributes to gliomagenesis remains to be investigated. Intriguingly, in a glioblastoma cell model we reproducibly observed high levels of cell death and additionally massive and rapid cell fusions upon PTPN13 shRNA-mediated knock-down (our unpublished data). This is not seen following depletion of PTPRZ-B [125]. The speed at which cell fusions occur rules out that the underlying mechanism reflects PTPN13’s contribution to cytokinesis [148]. The resulting large cell fusions rather are reminiscent of the rare Giant Cell Glioblastoma subtype [149] that is characterized by giant cells containing multiple nuclei [150]. Independent of its potential to bind to the PTPRZ-B C-terminus, it will be worthwhile to examine PTPN13 levels in Giant Cell Glioblastoma samples. 

About one-third of the glioblastomas carry inactivating mutations in the gene encoding PTEN. Interestingly, the PTEN C-terminus (“TKV”) is bound by many of the same PDZ domain-containing proteins that bind to PTPRZ-B (Figure 4E), including PSD95, MAGI-1 and PTPN13 [123]. In a recent study on conserved potential synthetic lethal gene pairs (meaning that inactivation of one of the genes in the pair will not kill the cell because the other will compensate, but inhibiting both genes does kill the cell) the combination *PTPRZ1*–*PTEN* was amongst the 20% most likely predictions [151]. Although *PTEN* and *PTPRZ1* are cancer modulating genes in their own right, this synthetic lethal hypothesis sheds more light on the occurrence of PTEN deletion and PTPRZ-B upregulation within glioma specimens. Since PTEN and PTPRZ-B at least in part share their PDZ domain-mediated interactome, altered expression of one will modify hardwiring of the other PTP. PDZ domain-mediated PTEN recruitment to the cell membrane allows a more efficient dephosphorylation of phospholipids (Figure 2J and Figure 4D) [152]. PTPRZ-B on the other hand already resides at the cell membrane and, although it can affect AKT through Fyn [153], it may also impact on the PI3K pathway by keeping PTEN from its PDZ-based anchoring spots. This may well involve PTPN13. In addition, for the PDZ target sequence (“SSF”; [116]) in the glioblastoma-relevant PTPRT [122] knowledge on the glioblastoma PDZ interactome could provide new therapeutic avenues. 

However, there are more PDZ-based interactions that hold promise of proteinaceous drug development to treat glioblastomas. Recently, a bidentate molecule (113B7) that prevents the syntenin PDZ domain from binding targets like mutant EGFR (EGFRvIII) was shown to increase radiosensitivity and decrease growth of glioma tumors in vivo [154]. Then, there is the example of PTPN4, of which the enzyme activity is autoinhibited by its PDZ domain [67,68,69,70]. Upon ligand binding, the PDZ-mediated inhibition is released and PTPN4 no longer protects glioblastoma cells from apoptosis [66]. Given the poor prognosis that glioblastoma patients await, and considering that for decades no major treatment improvements could be heralded, one can only hope that drugs targeting specific PDZ-mediated PPIs will rapidly enter the clinic. Furthermore, having viral proteins that bind via their C-terminus to PDZ domain-containing PTP family members —such as the oncogenic HPV E6 [146,155]—to interfere with cellular functions, the application of cell-penetrating PDZ-binding peptides may also have bearing for the treatment of HPV-positive cancers [156].

## 6. Conclusions and Perspectives

For decades, PTP enzymes have been reluctant to attempts to specifically interfere with their functioning. Increased knowledge on their contributions to diseases and on the regulatory principles that govern their actions is now aiding the removal of their stigma of being undruggable. We argue that, apart from initiatives to specifically target the catalytic PTP domains, strategies that aim at protein interactions mediated by these enzymes, notably—but not solely—PDZ domain-based interactions, provide appealing routes to modify PTP functioning. 

Of course, there are many hurdles. Especially when dealing with PPIs, one should realize that any post-translational modification, including phosphorylation, may alter binding specificities of target proteins or their partners. Furthermore, PPI inhibitors are of the competitive type, meaning that bioavailability and the reaching of the required dosage in target tissues represent tremendous challenges. Finally, in view of the complex interaction networks built by the plethora of biomolecules in cells, the targeting of protein–protein interactions inevitably will mean that the displaced components may then associate with promiscuous other interacting partners, with potential novel complications. On the other hand, by drugging just a single specific interaction in a target protein’s complex social network, one may cut off negative effects attributed to that single interaction while leaving remaining, beneficial interactions untouched. The current, massive studies towards protein–protein interactions and protein regulatory mechanisms will fuel these attempts to expand the toolbox to regulate and correct protein behavior, including that of the protein tyrosine phosphatase enzyme family. High hopes are on antibody-type biologicals that bind to and act via the extracellular portion of receptor-type PTPs and on cell-penetrating peptide-like compounds that specifically interfere with intracellular PTP-protein interactions.
